# A gelatinous veil inside the abdomen of a pancreatic cancer patient. What lies beneath it?

**DOI:** 10.1002/ccr3.4353

**Published:** 2021-06-10

**Authors:** Michail Vailas, Nikolaos Benetatos, Ioannis Maroulis, Charalampos Kaplanis, Nikolaos Drakos, Francesk Mulita, Maria Sotiropoulou, Stylianos Germanos

**Affiliations:** ^1^ Department of Surgery University of Patras University Hospital of Patras Patra Greece; ^2^ Evangelismos General Hospital Athens Greece

**Keywords:** ascites, chylous, lymphoscintigraphy, lymphangiogram, pancreas, surgery

## Abstract

Chylous ascites following pancreatic surgery results from surgical damage to the cisterna chyli or tributaries, with an incidence in pancreatic surgery of 1.8%‐11%. Usually, conservative treatment is effective.

## Graphical abstract

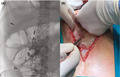

## 1 CASE DESCRIPTION

A 53‐year‐old patient presented to our department after the completion of neoadjuvant chemotherapy for a locally advanced pancreatic cancer. After multidisciplinary consultation, the patient underwent total pancreatectomy. Postoperatively, after the initiation of enteral feeding, the patient developed a high‐volume(2 L/d) drainage of milky yellow fluid in surgical drains.

**FIGURE 1:**
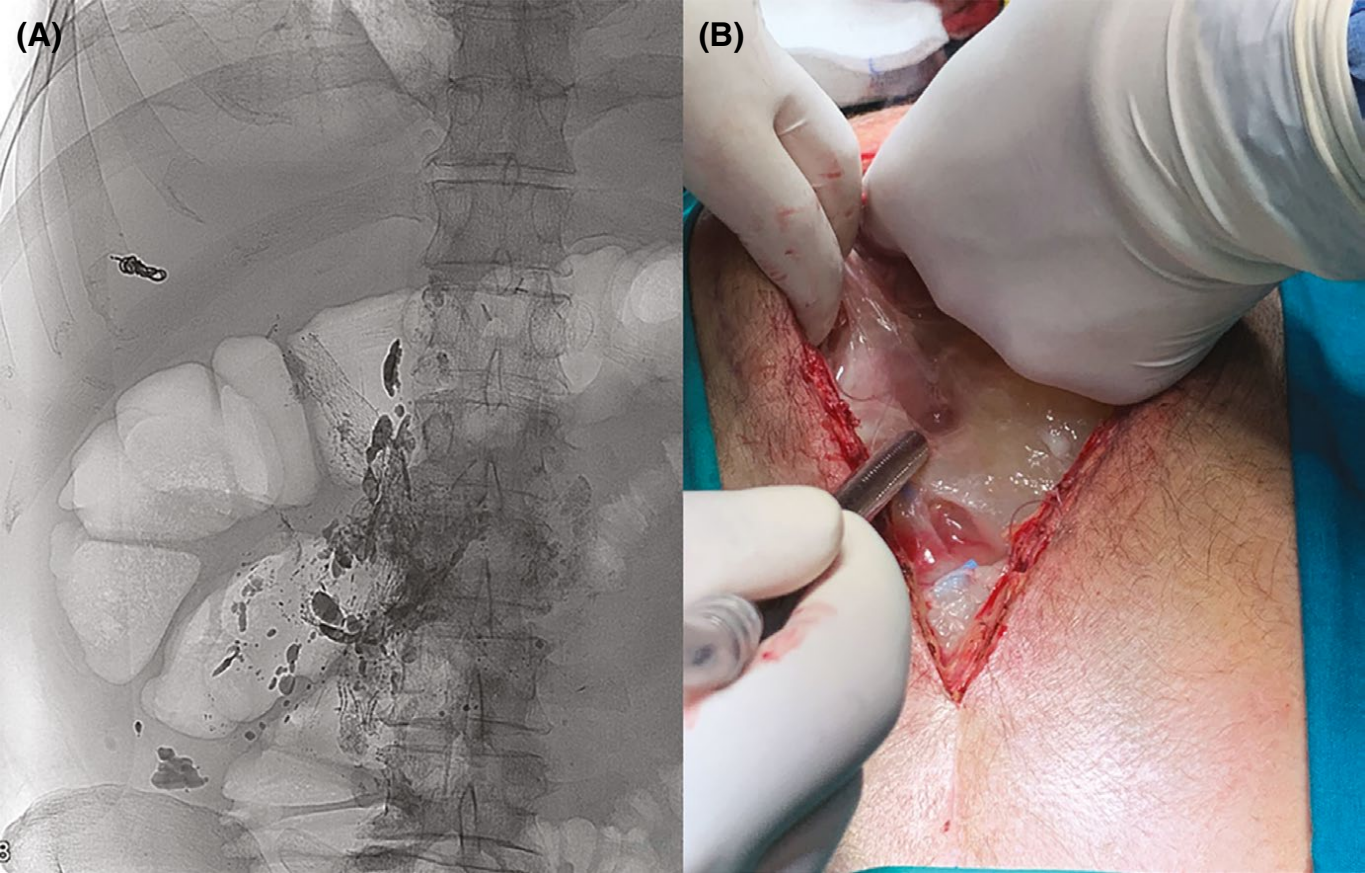
A, Lymphoscintigraphy and lymphangiogram showing a chyle leak adjacent to cisterna chyli. B, Intraoperative finding of chylous ascites with dense adhesions

Chylous ascites confirmed biochemically with triglyceride levels of the fluid exceeding 110 mg/dL. Conservative treatment with a combination of octreotide, a medium‐chain triglyceride diet, and total parenteral nutrition was followed. However, drainage output remained unaffected. Lymphoscintigraphy and lymphangiogram were performed showing the site of the leak adjacent to cisterna chili (Figure 1). Lipiodol was then slowly infused on both feet along with synthetic, biodegradable, and cyanoacrylate‐based glue in order to contain the leak. Following that, output was reduced (200 mL/d).

However, patient developed small bowel obstruction and underwent repeat laparotomy. Intraoperatively, the whole peritoneal cavity was found to be covered with a thick layer of gelatinous material (Figure 1). We performed extensive adhesiolysis and ligation of the leaking lymph vessels. Patient then followed an uneventful postoperative course.

Chylous ascites is a rare postoperative complication following major abdominal surgery. The skeletonization of vascular structures, as well as the more extensive retroperitoneal dissection, is considered risk factors for the development of chyle leak.^1^, ^2^ When diffused is associated with both a prolonged clinical course and increased mortality rates.^1^

## CONFLICT OF INTEREST

There are no conflicts of interest to declare.

## AUTHOR CONTRIBUTIONS

MV, NB, MS, CK, FM, and ND: contributed to the clinical data collection and prepared the case report. IM and SG: contributed to the design of the case report presentation and performed the final revision of the manuscript.

## ETHICAL APPROVAL

This report for a clinical image was conducted in accordance with the Declaration of Helsinki. The collection and evaluation of all protected patient health information were performed in a Health Insurance Portability and Accountability (HIPAA) complaint manner. A formal informed consent was obtained from the patient prior to the publication of this article.

## Data Availability

Data available on request from the authors.
